# Bodies in the Novel *Infinite Jest*

**DOI:** 10.3389/fpsyg.2021.539555

**Published:** 2021-09-09

**Authors:** Ana Chapman, Silvia Chapman, Stephanie Cosentino

**Affiliations:** ^1^Department of English, French and German, Faculty of Arts, University of Málaga, Málaga, Spain; ^2^Cognitive Neuroscience Division, Taub Institute for Research on Alzheimer's Disease and Gertrude H. Sergievsky Center, Columbia University Irving Medical Center, New York, NY, United States; ^3^Department of Neurology, Columbia University Irving Medical Center, New York, NY, United States

**Keywords:** literature, David Foster Wallace, body self-awareness, embodiment, cognitive science

## Abstract

This manuscript provides a literary analysis of the use of bodies in the novel *Infinite Jest* by David Foster Wallace. The novel describes a world where oversaturation of external stimulation leads to the perception of mind and body of self of an individual as prosthetic parts, malleable and deformed, wherein the mind fails to feel bodily sensations and characters experience a complete disconnectedness from the self and others. Indeed, the disembodiment of characters and sensations of disconnection leads them to a compulsive quest for connectedness through the use of masks, made-up feelings, mind–body hybrid pain, corporeal malleability, and prostheses. These portrayals of the disordered and disconnectedness between body and mind or self will be described and compared to clinical conditions characterized by a disconnection between mind and body and impaired body self-awareness. Through this exercise, we argue that the use of scientifically inspired pathologized bodies is a means of conveying the stance of Wallace on or criticism of the degradation of society through excessive entertainment.

## Introduction

Body and mind, and their relationship, have long been the object of interest across scientific, social, literary, and artistic fields. Descriptions, depictions, and interpretations of body and mind, in close connection to ownership and embodiment, are continuously evolving concepts representing a blending of ideas, figurative forms, and material conditions. Our lived experience is both nature- and culture-driven, giving rise to biological theories that include an analysis of social influences and by-products. The body has become an artifact increasingly represented as a double site of external and internal struggle. We argue that it is through an interdisciplinary perspective that we as humans attempt to understand the body as either the state of the self or an artistic or philosophical representation of an external force (i.e., society, divinity, etc.).

Throughout history, many corporeal representations have co-existed across philosophical, literary, and scientific perspectives. Images range from cave paintings wherein bodies were represented as simple mud figures focusing on action rather than form, to Haraway's (1991) cyborg or posthuman body, a hybrid creature composed of organisms and machines. Descriptions of the body in art have provided unique windows through which to view the state of philosophical and scientific quests that society undertakes to understand the role and purpose of the human body. Particularly, within literary work, the body and its connection to the mind have also been exploited as a medium to understand or express the social concerns of specific periods and cultures. Philosophers and artists, among others, have engaged in the discovery of the nature of the body while simultaneously incorporating knowledge and conceptions about the body derived from science, society, and religion (Fletcher, 1816; Shakespeare, 1980; Homer., 1987, 2003; Burton, 2001; Chaucer, 2003; Johnson, 2004; Wordsworth and Coleridge, 2013; Shelley, 2018 and in Dickens' work: Kryger, 2012). Using Illustration works depicting feminism, racism, and disability have also used the body as a means to represent these fights. As Robb and Harris (2013) state, the body enables us to “examine how historical circumstances create particular kinds of bodies, but [also] how particular kinds of bodies generate certain forms of history” (p. 4). From a scientific perspective, with the turn of the twentieth century, disciplines, such as neurology, neuroscience, neuropsychology, and psychology, flourished, and new theories of mind and body emerged. Diseases of the body were now understood through multifactorial lenses of neural underpinnings and cognitive and psychological processes. Common to all these disciplines is the newly found focus on the self and its disruption in the context of certain diseases.

This study aims to use the novel *Infinite Jest*, a twentieth-century novel wherein descriptions of bodily disturbances play a key part, to exemplify the intersectionality of artistic and scientific bodily perspectives reflective of the social and medical scientific context of the times. Although there have been many literary analyses of *Infinite Jest* such as those focusing on the themes of art vs. entertainment (Cioffi, 2000), addiction (Morris, 2001), solipsism and narcissism (Holland, 2013), and prosthetic description (Russell, 2010), among others, this study will focus solely on how bodies and body–mind connections are described in the novel. We suggest that contemporary (neuro)psychological and cognitive scientific works pertaining to body, mind, and self can be applied to understand the work of Wallace, and may even have served as sources of inspiration. Specifically, we propose that the physical distortions described by Wallace bring to mind contemporaneous neuroscientific models of disturbed disconnections between bodies and mind and body awareness, and in particular, how distortions in self-perception give rise to unique clinical phenomena. Descriptions of characters will be linked to theoretical and clinical constructs of embodiment (i.e., the “felt and conceptualized self” within own body of oneself; Crespi and Dinsdale, 2019), body image (i.e., the mental image or representation that individuals hold about their body shape, size, and form and the feelings associated with these; Slade, 1988), sense of ownership (i.e., the feeling that our body belongs to us; Gallagher, 2000), and sense of agency (e.g., the experience of being the agent that starts and controls an action; Moore and Fletcher, 2012).

This analysis widens the scope of how literature is a product of the mind and thus an interpretative device, rather than a verification of scientific theories of the mind; what literature aims to do, and what we attempt in this study, is not to “force” neuroscientific labels, but to examine how literary works can shed light on scientific perspectives that occupy a particular moment in history. This precisely connects to the field of cognitive literary studies that among other aspects analyze representations in literature from a cognitive lens (Hart, 2001; Spolsky, 2003; Richardson, 2010; Zunshine, 2010; Richardson and Spolsky, 2017). Particularly, we use a cognitive literary approach to analyze concepts of emotions, experience, and thoughts that are embedded, depicted, or encoded in literary works. Connections are made to embodied cognition (or the phenomenological and enactive streams of thought) where self-consciousness (of fictionalized characters, accounts of the author, or through the act of reading) arises from the living body. We will argue that through the intersectionality of clinical pathologies of the bodies within fictional characters, Wallace masterfully conveys his criticisms of the maladies of our current society, which seems to be becoming a normalized stance in society, abnormal embodiment and self.

## *Infinite jest*: Background of the Novel

Wallace is best known for his behemoth multilayered novel *Infinite Jest* and his nonfiction contributions. Published in 1996, *Infinite Jest* is a dense and complex 1,079-page novel with 388 endnotes depicting a dystopia originated from the overuse/overexposure and totalitarianism of the entertainment world in a country that resembles America.

Set in a near-future Boston, the novel examines the three main stories that crisscross and merge in many parts of the narrative. The three stories represent different sides of the entertainment world in which sport, addiction, and the media culture are analyzed in each intertwined narrative. First, tennis, one of the interests of Wallace, is represented through the Enfield Tennis Academy (E.T.A.) set up by Jim Incandenza, father of Orin, Hal, and Mario. Second, the main representation of addiction in the novel comes from the narration of the members of Ennet Alcohol and Drugs Rehabilitation House. This rehabilitation house establishes specific patterns of recovery through the commitment to a higher power. Third, the media is portrayed through a search of the *Assassins des Fauteuils Rollents* for Entertainment, a film, alternatively called Infinite Jest. The film, created by James Incandenza, is the ultimate stimulation that induces a catatonic death in its viewers. This film becomes the finest example of Wallace of how entertainment hinders action, willed choice, and experience through the destruction of body function, control, and sensations (basic bodily needs) to the extreme of possible death.

*Infinite Jest* is a book that works on obscurity and annularity, which results in an apparent non-concluding story of endless addiction and its effects. At the end of the novel, readers are left without much certitude of the course of events. The condition of Hal remains a mystery reflected in his unsettling smiling face and the uncertainty of his ability to speak as well as the condition of Don Gately at the hospital. Finally, the third story of *The Assassins*, the Canadian terrorists, ends with them on their way to Enfield Academy in search of deadly entertainment. Further, the novel is “fixated by its tools for chemical and electronic self-gratification [as it also] seems more prescient with the rollout of every new compulsively entertaining digital device” (Kalfus, 2010). It is a work that includes concerns and themes such as solipsism, language, terrorism, media, sport, addiction, self-reflexivity, irony, and politics. All events and characters intertwine in the discovery of what seems to reflect the loneliness sensed in each narrative, with crises of identity and self in an externally controlled and mediated world. Wallace points toward a “normality” through the inclusion of body abnormalities in the novel, wherein bodies are represented as fragmented and disconnected from an individual oneself and lacking a sense of agency. This affects the perception of the reader and it is seemingly used by the author as a tool to illustrate how society is leading toward this figurative mental illness as the “normalized” stance.

## Reflections on Neuroscience in *Infinite jest*

With the “cognitive turn” beginning in the 1990s, we can find a corresponding connection between literary works such as *Infinite Jest* with neuroscientific research. Cognitive interpretations of literary texts have a wide range of practice, values and perspectives, and have brought much discussion of the connection between both literature and neuroscience (e.g., effect on mind of the readers, interpretation of the text, etc). Caracciolo (2016) considers that a literary work “can serve as application or illustration of a scientific theory” but “evidence [cannot be found] for a particular view of the mind in a literary text, because textual evidence is different from scientific evidence” (pp. 192–193). That being said, fictional works, in general, then must be considered as what they are, literary productions that attempt to analyze and apply different concepts for understanding particular aspects of society. Furthermore, literary criticism allows a diversity of interpretations which, in this particular case, are based on the emerging cognitive literary approach surrounding theories of the mind where experience and consciousness seem to arise from bodily sensations, emotions, and its dialectic relationship with its surroundings.

Our study intends to illustrate neuroscientific theories of the time. Indeed, Wallace seems to have held a major concern regarding how body and mind disorders, as a result of entertainment and current societal trends, affect the identity of the self (full catalog of his library is available at Harry Ransom Center) and has been described as having a “career-long fascination with consciousness” (Burn, 2012, p. 373). Wallace himself struggled to understand his mental states (depression, addiction, and suicidal attempts). It is publicly known that he was admitted for substance addiction in 1989 in the psychiatric hospital associated with Harvard University. His knowledge of medicine is displayed in the medical terms and chemical components of drugs described in the novel. The abundance of medical and pharmaceutical language together with the many instances of mental illnesses such as the following, become a window to observe neuroscientific knowledge at his time.

And so but since the old CBC documentary's thesis was turning out pretty clearly to be SCHIZOPHRENIA: BODY, the voiceover evinced great clipped good cheer as it explained that well, yes, poor old Fenton here was more or less hopeless as an extra-institutional functioning unit, but that, on the up-side, science could at least give his existence some sort of meaning by studying him very carefully to help learn how schizophrenia manifested itself in the human body's brain. that, in other words, with the aid of cutting-edge Positron-Emission Topography or “P.E.T.” technology (since supplanted wholly by Invasive Digitals, Orin hears the developmental psychology graduate student mutter to herself, watching rapt over her cup, unaware that Orin's paralytically awake), they could scan and study how different parts of poor old Fenton's dysfunctional brain emitted positrons in a whole different topography than your average hale and hearty nondelusional God-fearing Alber-tan's brain, advancing science by injecting test-subject Fenton here with a special blood-brain-barrier-penetrating radioactive dye and then sticking him in the rotating body-sized receptacle of a P.E.T. Scanner. (Wallace, 2006, p. 47)

It must be noted that Wallace was obsessed with language, and he carefully explored the use of the most specific and accurate wording possible. Furthermore, his work was published during the “Decade of the Brain” and the “cognitive turn” in literature, leading to a flourish of cognitive literary studies that bring the science of the mind into literature (Jackson, 2000, 2003; Crane, 2001; Hart, 2001; Zunshine, 2010, 2017; Caracciolo, 2016; Burke and Troscianko, 2017). Bearing this in mind, we can present an analysis of the body in the novel in connection to contemporary studies on body and mind as well as to highlight knowledge of Wallace about theories of the mind.

## Disconnections in Body and Mind

In *Infinite Jest*, the hedonistic search for pleasure in the overexposed mediated world influences lived experiences of the characters, which, we propose, are seen through disconnections of the physical body, as well as disconnections between physical body and mind.

Bodies that are incomplete or deformed pervade *Infinite Jest*. Descriptions of bodies are narrated through the depiction of separate, prosthetic, malleable, monstrous bodies challenging what one may consider the natural or classical body. Characters in the novel are represented as lopsided collections of parts or dissembled bodies, far from being coherent and unitary entities, they are anti-biological representations of lived bodies that will never reach satisfactory completion. Body parts are detached in the way Deleuze and Guattari (2004) define fractals, “aggregates whose number of dimensions is fractional rather than whole, or else with continuous variations” (p. 534). On the very first page, Hal identifies the people on the board as not being complete, akin to non-united bodies as if he were “surrounded by heads and bodies” (Wallace, 2006, p. 3). Wallace consciously focuses on limbs to produce an autonomous and disconnected sense of corporeality. Following analysis of Russell, limbs present exaggerated forms as a “tendency towards unequal distribution and “jagged seams” (Russell, 2010, p. 149). Tennis players are described as an assemble of fragmented body parts, as “most of the E.T.A. upperclassmen have these vivid shoe-and-shirt tans that give them the classic look of bodies hastily assembled from different parts of bodies, especially when you throw in the heavily muscled legs and usually shallow chests and the two arms of different sizes” (Wallace, 2006, p. 100). Even though exaggerated forms in sports can be considered normal in that context, continuous illustration of the novel of body disruptions sets the focus on these disruptions and possible effects on the self. Don Gately, an ex-oral-narcotics addict, has “a massive and almost perfectly square head” (p. 55). Charles Tavis is somewhat impaired because “the two sides of his face didn't quite go together” (p. 521). Joelle Van Dyne, also called Madame Psychosis, belongs to the Union of the Hideously and Improbably Deformed (UHID) and calls for all “the prosthetically malmatched … the convulsively Tourettic. The Parkinsonianly tremulous…The teratoid of overall visage…The in any way asymmetrical” (p. 192) on the radio every night. Detached or separate parts are also presented in dreams of Orin in which:

In the dream, it's understandably vital to Orin that he disengages his head from the phylacteryish bind of his mother's disembodied head, and he cannot. Last night's Subject's note indicates that at some point last night Orin had clutched her head with both hands and tried to sort of stiff-arm her, though not in an ungentle or complaining way (the note, not the stiff-arm). The apparent amputation of the Moms' head from the rest of the Moms appears in the dream to be clean and surgically neat: there is no evidence of a stump or any kind of nubbin of the neck, even, and it is as if the base of the round pretty head had been sealed, and also sort of rounded off, so that her head is a large living ball, a globe with a face, attached to his own head's face. (p. 46)

Through the creative process of Wallace, disturbances are also seen in the disconnected experiences of body and mind, in which individuals are unable to register or experience that they are in control of their actions (Fink et al., 1999). It is important to note that most of the bodily descriptions in the novel are described in the third person (even though it is omnisciently narrated) and that the interpretations laid out in this manuscript are through the lens of clinically documented distortions of the self. (i.e., first-person).

In a rare instance of the first-person description, one of the main characters, Hal Incandenza stands during an interview to explain his low academic achievement and soon realizes that his perceptions of his movements are quite different than those of his audience:

Please don't think I don't care. “I lookout. Directed my way is horror. I rise from the chair. I see jowls sagging, eyebrows high on trembling foreheads, cheeks bright-white. The chair recedes below me.

“Sweet mother of Christ,” the Director says.

“I'm fine,” I tell them, standing. From the yellow Dean's expression, there's a brutal wind blowing from my direction. Academics' face has gone instantly old. Eight eyes have become blank discs that stare at whatever they see.

“Good God,” whispers Athletics.

“Please don't worry,” I say. “I can explain.” I soothe the air with a casual hand.

Both my arms are pinioned from behind by the Director of Comp., who wrestles me roughly down, on me with all his weight. I taste floor.[…]And his arms.” You didn't see it, Tavis. His arms were—“flailing. This sort of awful reaching wriggle. Waggling.”

The group looking briefly at someone outside my sight. (Wallace, 2006, p. 12)

Though Hal attempts to communicate that “nothing is wrong,” indeed, he “lies there catatonic, staring” (p. 13).

From a neuroscientific perspective, these various disturbances can be likened to disconnections between body and mind that can rise to disturbances of “self-awareness” in which self-referential processing (i.e., processing information related to the self) is impaired, and indicates some form of disconnection between brain (i.e., what he personally thinks or feels) and behavior. Agency, the experience of being in control of an action or behavior, is a key neuroscientific concept in the novel. Agency is shaped by two significant aspects, fact and sense. Fact alludes to the component of agency in which one purposefully makes things happen by dint of neuroanatomy of voluntary action (i.e., I want to perform an action or accomplish a specific outcome within actions). On the other hand, the sense is “that I am the one who is causing or generating an action. For example, the sense that I am the one who is causing something to move, or that I am the one who is generating a certain thought in my stream of consciousness” (Gallagher, 2000, p. 15). In other words, it implies that the observed outcomes are a direct consequence of initiating and controlling our actions to influence the outside world and the notion of being aware of the outcome (i.e., a conscious experience). Sense of agency is intimately bound with notions of freedom and responsibility traits that give reason to why, if we feel the experience, we consider ourselves initiators of the acts (Haggard and Tsakiris, 2009; Nichols, 2011).

Farrer and Firth, among others, identify that “[a]gency has been assigned a key role in self-consciousness” (Farrer and Frith, 2002, p. 596), and that is why according to Gallagher (2000), self-consciousness is constituted in part by a “minimal self.” This direct self-awareness includes self-ownership or the sense that “it is my body that is moving” and self-agency, or the sense that “I am the initiator of the action and thus that I am causally involved in production of that action” (p. 16). In the normal experience of voluntary or willed action, the sense of agency and the sense of ownership coincide and are indistinguishable. In the absence of neural injury, somatosensory signals (e.g., where is my limb in space, what position is it in) align with the neural commands that generate movement (Blakemore et al., 1998). This translates to a feeling of “doing” connected to the brain activity that commands the body to move. If the outcome is predicted accurately, then the sense of agency is stronger (Wegner and Wheatley, 1999). This attenuation permits a distinction between active and passive movements and therefore attributes a sense of agency to actions of individuals. Frith (2005) asserts in “The Neural Basis of Hallucinations and Delusions” that one of the more extensively accepted explanatory models of these symptoms indicates a dysfunction in the “forward model” system (Frith, 1992), whose role consists in predicting the sensory consequences of actions. Due to damage to this comparator of predictions and consequences, individuals cannot discern whether they initiated action and thus conclude that it must be out of the control of an individual.

As fictional characters, Hal and others do not display classic or complete clinical disorders but rather features that represent aspects of these fascinating syndromes. These links can be found with regard to clinical syndromes which include distortions of agency, self-awareness, and body ownership. Disturbances in an agency are characteristic of certain psychiatric and neurological disorders. For example, deficits of sense of agency are common in patients with schizophrenia (Gallagher, 2005; Jeannerod, 2009). Somatic passivity phenomena (or delusions of alien control) (Oyebode and Davison, 1989) are a hallmark symptom of schizophrenia. The passivity phenomena in schizophrenia are defined as actions, thoughts, and sensations perceived to be under external forces that can be linked to an abnormal sense of agency (Georgieff and Jeannerod, 1998; Frith et al., 2000). Schizophrenia patients with these symptoms report a loss of clear boundaries between the self and others and think/feel that their thoughts and actions are controlled by external forces rather than belonging to them (Blakemore et al., 2002; Lindner et al., 2005; Shergill et al., 2005). Interestingly, for these types of patients, the degree of feeling in “self-touch is as high as the same touch applied by someone else” (Frith, 2005, p. 171). It is not only that they do not perceive it as self-created, but also, that it is intensified by the feeling that it comes from an external entity. In other examples such as that of research of Lindner et al. (2005), schizophrenic patients believed eye movement originated from external forces, a sensation that was linked to a possible “deficit in the perceptual compensation of sensory consequences of one's actions” (p. 1119). This description evokes examples of characters in the novel discussing a dubious sense of control. Dr. Rusk, the psychologist who no player understands says that “on the level of objects and a protective infantile omnipotence where you experience magical thinking and your thoughts and the behavior of objects' relation to your narcissistic wishes, the counterphobia presents as the delusion of some special agency or control to compensate for some repressed wounded inner trauma having to do with absence of control” (Wallace, 2006, p. 550).

Disconnection between body and mind also characterizes a host of neurologic syndromes in which aspects of self-awareness are impaired. Patients who have developed paralysis of one side of their body (i.e., hemiplegia) are often unaware/disconnected from their lost function and deny any motor difficulties as a result. This disorder is commonly referred to as anosognosia, which translates to $\overset{∙}{\alpha }$- a-, “without,” νóσoς nosos, “disease” and $\gamma \nu \tilde{\omega }\sigma \iota \varsigma$ gnōsis, “knowledge” (Jenkinson et al., 2011; Mograbi and Morris, 2018). One of the most salient delusive phenomena is known as somatoparaphrenia (e.g., patient ascribes ownership of the limb to another person) (Gerstmann, 1942). Following Gerstmann's (1942) definition, for this syndrome to occur, a patient must have (i) acquired contralateral motor deficits, (ii) unawareness of such deficits, and (iii) delusional beliefs regarding the limbs affected by these deficits (Feinberg and Venneri, 2014). These delusional beliefs are specific to body ownership defined by Jenkinson et al. (2018) as the “sense, feeling or judgement that the body belongs to me and is ever present,” while other delusions such as *asomatognosia* (e.g., patient behaves as if their limb does not exist) typically entail delusions of *existence, visual self-reflection, and sense of belonging* of the contralateral limb (Jenkinson et al., 2018). Other disturbances can be observed as unilateral spatial neglect following brain injury (Vallar, 1998). Neglect specific to own body of an individual, also known as personal neglect (e.g., Pizzamiglio et al., 1989; Guariglia and Antonucci, 1992; McIntosh et al., 2000; Baas et al., 2011), has been proposed to reflect predominantly right-sided neuroanatomic compromise, including regions such as the postcentral and supramarginal gyri of the parietal cortex (Committeri et al., 2007, 2018). In this disorder, the patient is unable to direct their attention to one side of their body. The extent of this deficit is manifested in everyday activities, such as dressing or grooming, in which patients neglect to dress, brush their hair, or apply make-up on the contralesional side of their bodies (Caggiano and Jehkonen, 2018). Interestingly, anosognosia, asomatognosia, and neglect commonly co-occur and neglect has previously been proposed as an underlying mechanism for anosognosia (Cutting, 1978; Starkstein et al., 1992; Jehkonen et al., 2006). However, anosognosia and accompanying delusions are considered distinct from personal neglect. Indeed, dissociations of these disorders have been reported, and while patients with anosognosia can have delusions of ownership of the contralesional part of their body (e.g., somatoparaphrenia), neglect patients will acknowledge ownership if their attention is properly directed to their contralesional limbs (Bisiach et al., 1986; House and Hodges, 1988; Small and Ellis, 1996; Berti et al., 2005; Jenkinson et al., 2018). These clinical syndromes of body self-awareness may have inspired Wallace in the process of writing *Infinite Jest*. Particularly, one may see neglect as epitomized through the Entertainment film. Viewing the film ceases any corporal attention leading to a possible vegetative state or even death.

Disturbances in the mind–body connection in the novel invite comparison to neurologic disorders in which expression of affect through motor control, particularly through facial expressions, become impaired. Hal, for instance, has lost the ability to express through his body any feelings or sensations. The narration describes that “[f]or a brief moment that Hal will later regard as completely and uncomfortably bizarre, Hal feels at his own face to see whether he is wincing” (Wallace, 2006, p. 342). These anomalies of expression can be observed across multiple clinical disturbances. One such disorder is Parkinson's disease (PD) and atypical Parkinsonian disorders such as progressive supranuclear palsy (PSP). PD, a neurodegenerative disorder characterized by neuronal loss in the substantia nigra (Olanow et al., 2009), manifests in motor disturbances such as bradykinesia, rigidity, tremor, and postural instability (Gelb et al., 1999), while main symptoms of PSP are ocular motor dysfunction, postural instability, akinesia, and cognitive dysfunction (Höglinger et al., 2017). Both PD and PSP patients often show a loss of spontaneous and voluntary facial expressions, commonly referred to as *masked faces* (Buck and Duffy, 1980; Katsikitis and Pilowsky, 1988, 1991; Borod et al., 1990; Saku and Ellgring, 1992; Brozgold et al., 1998; Bowers et al., 2006; Marsili et al., 2014). These deficits can lead to incongruent and reduced/absent facial expressions such as the ones described by Wallace in *Infinite Jest*. Indeed, as opposed to healthy individuals, patients with PD and PSP experience difficulty in expressing facial expressions. These impairments are hypothesized to underlie the degeneration of brain networks that include structures, such as the amygdala and basal ganglia known to be key in emotion processing and motor action (e.g., Davidson et al., 2000; Gross and Thompson, 2006; Aron et al., 2014).

## Embodiment in the Novel

*Infinite Jest* seems to determine/reflect how the environment affects perception and self-awareness, in the sense of “here-ness” of characters at a given moment, and feeling/bodily sensations exist for a particular experience. Overall, a wide range of disruptions (in the vast majority related to emotions and bodily sensations/perceptions) of the novel can be interpreted as leading to the idea that to experience correct self-awareness, there is a need to connect these cognitive processes to a more grounded or lived experience of self. That is, as the narrative is fragmented (although connected in the paradigm of bodily disruptions and lack of correct affective framing), it leaves the question open to how characters can have a complete experience in light of the overpowering technological or entertainment industry. Experiences need to be studied from the disconnection of the organism from its environment. In this case, Wallace reflects on the loneliness of characters that have lost the sense of belonging or communicating with their environment (not only the surroundings but also others). The body in the novel lacks situatedness and the correct arousal of the affective component (as in a dialogue with its surroundings).

The novel is about the sadness, loneliness, or pain of not being connected. As previously discussed, there is a sensation of disconnection with the surroundings that are essential for a complete sense of self-awareness of oneself. The following section is particularly relevant for its intimate discussion on core sections of awareness:

Hal, who's empty but not dumb, theorizes privately that what passes for hip cynical transcendence of sentiment is really some kind of fear of being really human since to be really human (at least as he conceptualizes it) is probably to be unavoidably sentimental and naive and goo-prone and generally pathetic, is to be in some basic interior way forever infantile, some sort of not-quite-right-looking infant dragging itself analytically around the map, with big wet eyes and froggy-soft skin, huge skull, gooey drool.” (Wallace, 2006, p. 694)

The narrative suggests that the disconnection of the “egocentric” or “inner source-point,” (Maiese, 2016, p. 10) that is, the “internal self” to bodily sensations (“sentiment and need”), and the disturbances in the situated bodily experience within its environment (“empty mask, anhedonia”) are what seems to lie behind self-detachment in the novel. Indeed, lived experiences described in *Infinite Jest* are portrayed as deviated/distorted by disembodied bodies and disturbances perception (connects to theories of the phenomenology of Merleau-Pounty).

The most intimate or personal sections include the influence of embodied cognition or phenomenological description (mainly through tennis and Kate Gompert) demonstrating the importance of the body in experience or for self-awareness: “I was in my body. My body and me were one … my well-armed hand was the secretary of my mind, lithe and responsive and *senza errori*, because I knew myself as a body and was fully inside my little child's body out there” (Wallace, 2006, p. 165).

## Conclusion

This study has offered a constructive analysis to demonstrate how literature is a well-established vehicle to interpret or illustrate clinical studies/knowledge as well as current social and technological developments of a given time. Societal fascination over the self is also seen through the exploration and discussion of status of the individual in connection to these developments throughout history. Social issues then seem to align with new concepts and discoveries in scientific, technological, and medical fields. One way of interdisciplinary inquiry that reflects upon social curiosity or concerns is through literature as we have attempted to show in this study.

First of all, it has discussed renowned literary authors who have included medical knowledge and discussion in their works throughout history. Second, it has shown parallelism between the fictionalized world in *Infinite Jest* and some major aspects regarding current neuropsychological research on the role of body awareness in shaping the self. In the world of the novel, the state of the body is depicted as malleable, fractioned, deformed, and disconnected from the mind, which gives its characters an unstable sense of self. This is also translated into a dubious sense/feeling of embodiment that has a major role in representing and attributing body and actions of an individual to the self of an individual. By contrasting the representation of bodies and sense of agency in the novel with disorders, such as anosognosia, anorexia, schizophrenia, disembodiment, and lack of sense of agency, we have demonstrated how literature can also supply a resource for examples of body and self not only in a literary way but also reflecting a medical one. These examples also parallel current social issues on the overuse of entertainment and technology, addiction, and the dependency on these external stimuli for identity and self. In this manner, the discussion of these concerns seems to become intertwined with current neuroscientific discoveries observed in this literary text. Knowledge of Wallace on psychological theories detected in his novel has allowed us to explore and connect body self-awareness and mind issues of the period. From a literary standpoint, the adaptation of disorders in body self-awareness and the self also enable Wallace to expose how entertainment and the contemporary way of living has an impact on body self-awareness and the self, thus demonstrating an illustrative relationship between the social, literary, and medical fields of a historical period.

The novel develops a vision of a society whose pursuit of pleasure was shutting itself off from true feeling and experience. A society that was fragmented and not unified/connected but rather constituted by disconnected/self-detached and solipsistic individuals that only share the common ground of alienation and addiction to entertainment. Wallace sets a general medicalized framework (inserting many references to drugs and mental disorders and body disturbances), addiction, and the entertainment industry to explore human emotions and experience. Rather than providing answers or a general diagnosis and “recovery,” the dystopian plot opens up to a more embodied ground for self-awareness. As observed in the literary work, “your self is not only your head but body” (Wallace, 2006, p. 272); Self-awareness then is connected to lived time, the living body and its connection and responses to the environment which emphasizes subjective and lived experience observed in embodied cognition models and in the novel, Wallace shows dislocation/alienation from the globalized but calls for the sincerity of experience. The novel does not “just diagnose a malaise. It proposed a treatment” (Max, 2012, p. 214) from lack of awareness moving away from first-wave cognitive studies of diagnosis as neurological reactions and thus getting closer to understanding through “fiction … what it is to be a fucking *human being*” (McCaffery, 1993, p. 131).

## Author Contributions

AC took the lead in writing the manuscript. SCh helped shape analysis and provided critical feedback. SCo supervised and provided feedback on the final versions of the manuscript. All authors contributed to the article and approved the submitted version.

## Conflict of Interest

The authors declare that the research was conducted in the absence of any commercial or financial relationships that could be construed as a potential conflict of interest.

## Publisher's Note

All claims expressed in this article are solely those of the authors and do not necessarily represent those of their affiliated organizations, or those of the publisher, the editors and the reviewers. Any product that may be evaluated in this article, or claim that may be made by its manufacturer, is not guaranteed or endorsed by the publisher.
